# Pelvic Floor Dysfunction and Manometric Features in Pediatric Solitary Rectal Ulcer Syndrome

**DOI:** 10.3390/jcm15062140

**Published:** 2026-03-11

**Authors:** Nihal Uyar Aksu, Altay Çelebi, Ayşen Uncuoğlu

**Affiliations:** 1 Division of Pediatric Gastroenterology, Hepatology and Nutrition, Kocaeli University Faculty of Medicine, 41001 Kocaeli, Türkiye; aysen.uncuoglu@kocaeli.edu.tr; 2 Department of Gastroenterology, Kocaeli University Faculty of Medicine, 41001 Kocaeli, Türkiye; altaycelebi@kocaeli.edu.tr

**Keywords:** pediatric solitary rectal ulcer syndrome, dyssynergic defecation, pelvic floor dysfunction, rectal bleeding, constipation, biofeedback therapy

## Abstract

**Background/Objectives:** Solitary rectal ulcer syndrome (SRUS) is a rare benign disorder presenting with rectal bleeding, straining, and mucosal discharge. Its pathogenesis likely involves pelvic floor dysfunction, particularly dyssynergic defecation. Although studied in adults, pediatric data—specifically anorectal manometry (ARM) findings—remain limited. We aimed to evaluate dyssynergic defecation in pediatric SRUS using ARM and analyze associated clinical, endoscopic, histopathological, and treatment data. **Methods:** A retrospective study of 24 children with biopsy-proven SRUS diagnosed between 2016 and 2024 was conducted. Clinical symptoms, colonoscopic, histopathological, treatment, and outcome data were reviewed. ARM was performed in 20 patients unresponsive to conservative treatment to assess anal pressures, rectal sensation, rectoanal inhibitory reflex, and balloon expulsion. **Results:** The median age was 13 years, with male predominance. Rectal bleeding was the most common symptom (95.8%). Colonoscopy revealed predominantly solitary ulcerative lesions 5–10 cm from the anal verge. Dyssynergic defecation was detected in 60% of patients, and only 25% could expel the balloon. Resting anal pressures were lower than reference values. Treatments included diet, laxatives, and topical agents, with partial or complete clinical response in approximately 60% of patients after 12 months. **Conclusions:** Pediatric SRUS is strongly associated with dyssynergic defecation. More pediatric-specific manometric studies are needed to optimize diagnosis and guide targeted therapies.

## 1. Introduction

Solitary rectal ulcer syndrome (SRUS) is a benign condition diagnosed based on a combination of clinical symptoms and endoscopic and histopathological findings. Its estimated prevalence in adults is approximately 1:100,000. Although its prevalence in children remains unknown, SRUS is widely regarded as a rare and underrecognized pediatric disorder [1].

Patients commonly present with rectal bleeding. Other reported symptoms include tenesmus, straining during defecation, mucoid rectal discharge, abdominal and perianal pain, constipation, and diarrhea. Ulceration, erythema, and polypoid lesions are the characteristic macroscopic findings of SRUS on endoscopy, and histopathological evaluation is required to confirm this diagnosis. Typical histopathological features include fibromuscular obliteration of the lamina propria by fibroblasts and muscle fibers originating from the muscularis mucosa, thickening of the muscularis mucosa, and glandular crypt abnormalities [2].

However, the etiology of SRUS remains unclear. Proposed mechanisms include rectal digitation, rectal prolapse, intussusception, rectal hypersensitivity leading to excessive straining, and pelvic dyssynergia. Among these potential mechanisms, pelvic dyssynergia—characterized by abnormal contraction of the puborectalis muscle in response to increased intra-abdominal pressure during straining—is the most widely emphasized in the literature. This abnormal contraction causes the anterior rectal wall to press against the upper anal canal, leading to local ischemia, edema, and ulceration [3,4]. Anorectal manometry (ARM) and balloon expulsion testing are recommended tools to evaluate defecation disorders, including dyssynergic defecation [5,6]. In children, ARM protocols and normal values are less well standardized than in adults; the British Society of Paediatric Gastroenterology, Hepatology and Nutrition-Motility Working Group (BSPGHAN-MWG) recently proposed consensus recommendations and suggested using adult reference values for children older than 12 years, with adaptation based on published pediatric cohorts [5]. Despite the increasing use of ARM in pediatric defecation disorders, there remains a lack of pediatric SRUS cohorts that include comprehensive manometric assessments and balloon expulsion tests. This gap hampers a mechanistic understanding of SRUS in children and prevents the development of targeted therapies such as pelvic floor biofeedback. Therefore, this study aimed to assess the role of dyssynergia in pediatric patients with SRUS. We also examined the clinical history, symptoms, colonoscopic and histopathological findings, and medical treatments of SRUS in this population. To our knowledge, this is the first pediatric SRUS study to apply a standardized ARM protocol interpreted using BSPGHAN-MWG consensus recommendations, report anorectal pressure profiles together with rectal sensory thresholds, and systematically evaluate balloon expulsion performance in a biopsy-proven pediatric SRUS cohort. In this regard, we aim to better define the manometric phenotype of pediatric SRUS and to support the role of pelvic floor dysfunction in its pathogenesis.

## 2. Materials and Methods

This retrospective study was approved by the Clinical Research Ethics Committee of the Kocaeli University Faculty of Medicine (Approval No: 2024/385; Approval date: 20 September 2024).

### 2.1. Study Design

We retrospectively identified all patients aged <18 years who were diagnosed with SRUS between January 2016 and June 2024 at the Department of Pediatric Gastroenterology, Kocaeli University Faculty of Medicine Hospital. Inclusion criteria were (1) presence of rectal lesions on colonoscopy compatible with SRUS (ulcerative, polypoid, or erythematous lesions confined to the rectum) and (2) histopathological confirmation of SRUS, defined as fibromuscular obliteration of the lamina propria with splaying of smooth muscle fibers from the muscularis mucosa and characteristic crypt abnormalities [2,3].

Exclusion criteria were (1) known inflammatory bowel disease, celiac disease, or other organic gastrointestinal disease; (2) previous colorectal surgery; (3) neurologic or spinal disorders affecting anorectal function; (4) congenital anorectal malformations; and (5) incomplete clinical or endoscopic records.

All patients were initially evaluated with a standardized diagnostic workflow, including detailed clinical history, physical and perianal examination, digital rectal examination, laboratory tests when indicated, and colonoscopy with biopsies. Functional constipation was diagnosed according to Rome IV criteria.

All patients underwent colonoscopy using standard pediatric colonoscopes. The procedure was performed under intravenous sedation according to institutional pediatric sedation protocols. Rectal lesions were carefully inspected, and their number (solitary or multiple), morphology (ulcerative, polypoid, or erythematous), and distance from the anal verge were recorded. Multiple biopsies were obtained from the lesion(s) and, when indicated, from adjacent normal-appearing rectal mucosa. Histopathological confirmation of SRUS was required for inclusion in the study.

An experienced gastrointestinal pathologist reviewed all slides. The diagnosis of SRUS was based on the presence of characteristic features, including (1) fibromuscular obliteration of the lamina propria with proliferation of fibroblasts and smooth muscle fibers extending from the muscularis mucosa; (2) thickening and splaying of the muscularis mucosa; and (3) glandular crypt abnormalities such as distortion, branching, and mild crypt hyperplasia [2,3].

Anorectal manometry was offered to patients with persistent symptoms despite at least 2 months of optimized conservative therapy, including toilet training, a high-fiber diet, avoidance of excessive straining and digital evacuation, and appropriate laxative and topical therapy.

Data collected included patient age, sex, clinical presentation, duration of symptoms, colonoscopic, histopathologic, and anorectal manometric findings, laboratory results, treatments, and treatment outcomes.

Symptom duration was defined as the time interval from symptom onset to the diagnosis of SRUS.

Treatment adherence was assessed at each clinic visit based on caregiver and patient report and was categorized as adequate when ≥80% of prescribed measures were followed.

Clinical outcomes were evaluated at approximately 12 months after initiation of treatment. ‘Complete response’ was defined as complete resolution of rectal bleeding and other SRUS-related symptoms. ‘Partial response’ was defined as a ≥50% reduction in symptom frequency or severity (including rectal bleeding) compared with baseline. ‘No response’ was defined as <50% improvement in symptoms.

### 2.2. Anorectal Manometry

ARM was performed in patients who did not respond to at least 2 months of dietary modification, avoidance of excess straining, laxatives, topical sucralfate, or mesalazine. The procedure was performed in fully awake patients without sedation, in the left lateral position, using a 4-channel air-charged conventional catheter (MMS, Solar System, Enschede, The Netherlands). All patients received one sodium phosphate enema (118 mL) at least 1 h before the study. Anal sphincter function, rectoanal reflex activity, rectal sensation, changes in anal and rectal pressures during attempted defecation, rectal compliance, and balloon expulsion test (BET) performance were measured.

Resting and squeeze pressures, rectal sensation thresholds, and the rectoanal inhibitory reflex (RAIR) were interpreted using the pediatric reference ranges proposed by Banasiuk et al. [7] for children older than 12 years, as adopted by the BSPGHAN-MWG [5].

Resting anal pressure was determined by averaging readings from pressure sensors in the anal canal over a minimum duration of 30 s, following a variable adaptation period typically ranging from 1 to 5 min [5,6].

Squeeze pressure was assessed following a voluntary squeeze contraction of the anal muscles as forcefully as possible for a duration of 15–20 s. To get the best squeeze, this was done twice. The maximum pressure measured during a contraction session lasting more than two seconds was specified as maximum anal squeeze pressure. Rectal sensation thresholds were assessed using isovolumetric balloon-air inflation, during which the rectal balloon was gradually inflated with air. The first sensation volume was defined as the minimum balloon volume at which the patient perceived any rectal sensation. The desire to have a bowel movement that lasts less than fifteen seconds is referred to as the desire to defecate. The urge to defecate is characterized by an intense desire for a bowel movement, necessitating an immediate cessation of activities to get to the restroom [8]. The maximum tolerable volume was obtained when the patient was unable to tolerate further rectal distension.

The rectoanal inhibitory reflex (RAIR) was assessed by rapid inflation and deflation of the intrarectal balloon with 10–60 mL of air. The reflex was considered present when rectal distension produced a decrease of >20% in anal pressure relative to the resting value.

Dyssynergic defecation was identified when two criteria were fulfilled: the presence of a dyssynergic pattern during attempted defecation on anorectal manometry and an abnormal balloon expulsion test [9]. In accordance with BSPGHAN-MWG consensus and adult criteria adapted for children > 12 years [5] dyssynergia was defined as: (1) adequate intra-abdominal pressure with high anal sphincter pressure, (2) poor push with high anal sphincter pressure, (3) adequate intra-abdominal pressure with no decrease in anal sphincter pressure, (4) poor push with no decrease in anal sphincter pressure.

Balloon expulsion testing (BET) was performed immediately after manometry using a 4 cm latex disposable balloon attached to a 2 mm tube, filled with 50 mL of warm water. The balloon was inserted with the patient in the left lateral position. Then, sitting on a commode, the child was instructed to expel the balloon in privacy. Failure to expel the balloon within 2 min was considered abnormal. This protocol is consistent with previously published adult and pediatric studies [10,11].

### 2.3. Statistical Analysis

All statistical analyses were performed using IBM SPSS for Windows version 29.0 (IBM Corp., Armonk, NY, USA). The Shapiro–Wilk test was used to assess normality. Continuous variables with normal distribution were presented as mean ± standard deviation (SD), while non-normally distributed variables were presented as median and interquartile range (IQR). Categorical variables were presented as frequencies and percentages. All statistical analyses were reviewed by a biomedical statistician from the Department of Biostatistics and Medical Informatics to ensure accuracy and appropriate methodology

## 3. Results

### 3.1. Demographic and Clinical Characteristics of Patients

A total of 24 patients with biopsy-proven SRUS were included in the analysis. The cohort comprised 20 boys (83.3%) and 4 girls (16.7%). The median age at presentation was 13 years (IQR: 11–15 years), and the median duration of symptoms before diagnosis was 21 months (IQR: 5.25–39.75 months) (Table 1).

Rectal bleeding was the most common presenting symptom, reported in 23/24 patients (95.8%).

### 3.2. Findings at Colonoscopy

According to the colonoscopic findings, 16 patients (66.7%) had a single lesion, and the remaining patients had multiple lesions. All lesions were located 5–10 cm from the anal verge. Ulcerations were observed in 22 patients, and two patients had polypoid lesions. Rectal prolapse was documented in two patients (8.3%).

### 3.3. Anorectal Manometric Findings

Anal manometry was performed in 20 patients. Patients who underwent ARM tended to have a longer duration and greater severity of symptoms, as they were selected based on non-response to conservative treatment. However, no significant differences in baseline clinical characteristics were observed between the ARM and non-ARM groups.

The interval between treatment initiation and anal manometry was 5.1 months (SD ± 3.08). The mean resting anal pressure was 86.55 mmHg (SD ± 10.87). The maximum squeeze pressure was 111.5 mmHg (IQR: 90.25–164.75). The first rectal sensation volume was 58.5 cm^3^ (SD ± 24.5), and the maximum tolerable volume was 156 cm^3^ (SD ± 46.27). The RAIR was intact in all patients. Dyssynergic defecation was observed in 60% of patients (Table 2).

Of the 20 patients who underwent ARM, 20 also completed the BET. Only 5/20 patients (25.0%) were able to expel the balloon within 2 min, whereas 15/20 (75.0%) had an abnormal BET. None of the patients exhibiting dyssynergic defecation were capable of expelling the balloon.

### 3.4. Treatment of Patients

All patients received standardized first-line management consisting of toilet training, education to avoid excessive straining and digital evacuation, a high-fiber diet, and, when indicated, osmotic or stimulant laxatives. Topical therapy included sucralfate enemas, mesalazine enemas, or a combination, prescribed according to symptom severity and endoscopic findings.

Laxatives were prescribed in 15/24 patients (62.5%). Sucralfate enemas were used in 15/24 (62.5%), mesalazine enemas in 3/24 (12.5%), and a combination of both in 6/24 (25.0%). Treatment adherence was adequate in 14/24 patients (58.3%). At approximately 12 months of follow-up, 2/23 evaluable patients (8.7%) achieved complete clinical response, 12/23 (52.2%) had a partial response, and 9/23 (39.1%) had no response.

## 4. Discussion

The most distinctive finding of our study is the high prevalence of pelvic floor dysfunction in pediatric SRUS: 60% of children demonstrated dyssynergic defecation on ARM, and all of these children had abnormal BET. These rates are comparable to those reported in adult SRUS cohorts, where dyssynergic defecation and fecal evacuation disorders have been observed in 25–82% of patients [10,11]. Sharma et al. [12] found abnormal BET results in 53% of patients with SRUS and 26% of healthy controls. Anal relaxation and BET findings were significantly abnormal in patients with SRUS compared with healthy controls (53% vs. 20%) in another study highlighting the strong association between SRUS and defecatory dysfunction [13]. Our data suggest that a similar pathophysiological link exists in children, supporting the view that dyssynergic defecation is a key mechanism rather than a coincidental finding.

Despite numerous hypothesized explanations, the pathophysiology of SRUS likely reflects a complex interaction of multiple factors rather than a single underlying cause [14]. SRUS may represent a final common pathway of several mechanisms that result in mucosal injury, such as trauma, ischemia, and abnormal defecation dynamics. The limited understanding of its pathophysiology continues to impede the development of effective and targeted treatment strategies.

ARM is a vital functional assessment tool for evaluating SRUS, offering essential insights into defecation dynamics, pressure profiles, rectal compliance, and sensory thresholds [15]. Moreover, it directly informs treatment by detecting functional abnormalities that can be targeted with therapies such as biofeedback. During treatment, manometry can also be used to monitor patients regularly and assess therapeutic response based on variations in manometric patterns [16]. However, the existing literature indicates that the absence of pediatric-specific SRUS manometry studies constitutes a substantial gap that limits evidence-based treatment methodologies in children. In this study, 20 patients underwent ARM. Pediatric normative values for ARM are lacking. BSPGHAN-MWG recommends using adult reference values for children older than 12 years and adopts the values published by Banasiuk et al. [5,7]. Banasiuk et al. [7] reported the largest pediatric cohort evaluated using three-dimensional high-resolution ARM. Since conventional manometry was performed in our study, interpreting the results can be challenging because conventional ARM lacks standardized protocols and universally accepted normative values. Although the International Anorectal Physiology Working Group has published a standardized protocol—the London Protocol—to harmonize the reporting of anorectal physiology studies, normal pressure values still vary according to catheter type, sex, and age. Each center should therefore establish its own reference ranges.

As the median age of our patients was >12 years, the pediatric reference values for children > 12 years could be applied as it is recommended in the BSPGHAN-MWG consensus statement [5]. This approach was chosen due to the lack of pediatric-specific data and the applicability of adult reference ranges to older children. However, it is acknowledged that this may limit the interpretability of our findings due to potential discrepancies between pediatric and adult populations. The mean resting anal pressure and maximum squeeze pressure of our patients were below the normal values defined in the BSPGHAN-MWG consensus statement. On the other hand, first sensation volume and urge to defecate were higher than the reference values. Alterations in sensory function have also been reported in other studies. Compared with healthy controls, patients with SRUS demonstrated significantly decreased resting perineal pressures, increased anal electrosensory thresholds, and rectal hypersensitivity [17,18]. Reference values vary according to age, sex, body mass index, and testing methodology.

The median age of patients at presentation was 13 years. According to numerous pediatric studies, the median age at presentation ranges from 8 to 12 years, with the most affected children being older than 8 years [1,19]. There was a male predominance in our study, consistent with findings of other pediatric studies [1,20,21]. In contrast, SRUS affects both sexes relatively equally in adult populations.

In children, SRUS presents with a variety of symptoms that are consistent but nonspecific, making diagnosis challenging without a high index of suspicion. The most common presenting symptom was rectal bleeding (95.8%), which aligns with findings from other pediatric studies [1,22]. Excessive straining, which generates high intrarectal pressure and forces the anterior rectal mucosa against the contracting puborectalis muscle, is considered one of the mechanisms underlying SRUS. In this study, 58.3% of patients reported excessive straining, which is less frequent than in other pediatric studies [1,23]. This discrepancy may be attributed to patient embarrassment in reporting this symptom.

The median duration of symptoms was 21 months. This finding is consistent with that reported by Poddar et al., who described the largest pediatric cohort [1]. We believe that diagnosis was often delayed due to symptom overlap with other rectal disorders and limited awareness of the condition.

The name “solitary rectal ulcer syndrome” is considered a misnomer for several reasons: ulcers are present in only 40% of patients, a true solitary ulcer is found in only 20% of patients, and the condition may occasionally involve the sigmoid colon [13,24]. However, our findings support the disease name, as the lesions were confined to the distal rectum, which is typical, and were solitary in 66.7% of the patients.

Unlike other pediatric studies, our patients were observed over a protracted period of approximately 12 months [1,25]. We found that the overall response to toilet training, a high-fiber diet, and medical therapy was very low. This finding underscores that effective management of SRUS requires more than just behavioral modification and medication, owing to the contribution of pelvic dyssynergia. We were unable to assess the effects of biofeedback therapy on the treatment of SRUS. When combined with manometric monitoring, biofeedback therapy has shown promising results in adult patients with SRUS [26]. Research indicates that changes in anal resting pressure following biofeedback therapy may serve as important indicators of treatment efficacy. Functional evaluation with defecography also provides valuable insight into anorectal dynamics implicated in SRUS pathophysiology. Unfortunately, defecography was not performed at our hospital.

Although the study has reached its aim, it has several limitations. It was a retrospective, single-center study with a relatively small sample size. The absence of a healthy control group and the lack of defecography limit the generalizability of our findings. Furthermore, we were unable to remark on quantitative parameters since the pediatric normal values for air-charged conventional ARM are absent. In addition, we could not evaluate the efficacy of biofeedback therapy in our cohort. These limitations highlight the need for larger, prospective, multi-center studies to validate and expand upon our results.

The primary strength of this study lies in the anorectal manometric evaluation of pediatric patients with SRUS. We believe that this finding underscores the significance of dyssynergic defecation in the pathogenesis of SRUS. The observed correlation between dyssynergia and SRUS suggests that pelvic floor biofeedback plays a significant role in the management of these patients. Future studies employing larger cohorts of pediatric patients with SRUS, along with healthy controls, are warranted to evaluate the efficacy of biofeedback interventions for pediatric patients with defecatory dysfunction based on ARM findings.

## 5. Conclusions

The paucity of pediatric-focused SRUS manometry research represents a significant limitation in the development of evidence-based diagnostic and treatment strategies for children with this condition. This gap emphasizes the need for additional pediatric-specific studies to better characterize distinct manometric features, treatment modalities, and treatment responses in children with SRUS.

## Figures and Tables

**Table 1:** Demographics and symptomatic findings.

	*n* = 24
**Age at presentation (months)**	163.5 (136.25–186)
**Gender**	
Female	4 (17%)
Male	20 (83%)
**Duration of symptoms (months)**	21 (5.25–39.75)
**Symptoms**	
Abdominal pain	15 (62.50%)
Constipation	14 (58.30%)
Altered bowel habits	12 (50%)
Diarrhea	8 (33.30%)
Rectal bleeding	23 (95.80%)
Mucus per rectum	13 (54.20%)
Tenesmus	12 (50%)
Manual digital evacuation	8 (33.30%)
Excessive straining during defecation	14 (58.30%)
Rectal prolapse	2 (8.30%)
Results are presented as number (percentage) or median (IQR)	

**Table 2:** Anorectal Manometric Findings.

	*n* = 20
Anal canal resting pressure (mmHg)	86.55 ± 10.87
Maximum squeeze pressure (mmHg)	111.50 (90.25–164.75)
First rectal sensation (cm^3^)	50 (40–82.50)
Desire to defecate (cm^3^)	91 ± 31.44
Urge to defecate (cm^3^)	120 (100–160)
Maximum Tolerable Volume (cm^3^)	156 ± 46.27
Balloon Expulsion Test	
*Positive*	5 (25%)
*Negative*	15 (75%)
Rectoanal inhibitory reflex	
*Positive*	20 (100%)
Dyssynergic defecation	
*Positive*	12 (60%)
*Negative*	8 (40%)
Results are presented as number (percentage), mean ± SD, or median (IQR)	

## Data Availability

The data presented in this study are available on request from the corresponding author. The data are not publicly available due to privacy of the individuals and for institutional ethical restrictions.

## Abbreviations

The following abbreviations are used in this manuscript:

SRUS	Solitary rectal ulcer syndrome
ARM	Anorectal manometry
BSPGHAN-MWG	British Society of Pediatric Gastroenterology Hepatology and Nutrition-Motility Working Group
BET	Balloon expulsion test
RAIR	Rectoanal inhibitory reflex

## References

[1] Poddar U., Yachha S.K., Krishnani N., Kumari N., Srivastava A., Sen Sarma M. (2020). Solitary Rectal Ulcer Syndrome in Children: A Report of 140 Cases. J. Pediatr. Gastroenterol. Nutr..

[2] Chiang J.M., Changchien C.R., Chen J.R. (2006). Solitary rectal ulcer syndrome: An endoscopic and histological presentation and literature review. Int. J. Color. Dis..

[3] Sharara A.I., Azar C., Amr S.S., Haddad M., Eloubeidi M.A. (2005). Solitary rectal ulcer syndrome: Endoscopic spectrum and review of the literature. Gastrointest. Endosc..

[4] Ertem D. (2012). An Overlooked Entity in Children with Rectal Bleeding: Solitary Rectal Ulcer. J. Pediatr. Gastroenterol. Nutr..

[5] Athanasakos E., Cleeve S., Thapar N., Lindley K., Perring S., Cronin H., Borrelli O., Mutalib M. (2020). Anorectal manometry in children with defecation disorders BSPGHAN Motility Working Group consensus statement. Neurogastroenterol. Motil..

[6] Strisciuglio C., Banasiuk M., Salvatore S., Borrelli O., Staiano A., Van Wijk M., Vandenplas Y., Benninga M.A., Thapar N. (2022). Anorectal Manometry in Children: The Update on the Indications and the Protocol of the Procedure. J. Pediatr. Gastroenterol. Nutr..

[7] Banasiuk M., Banaszkiewicz A., Dziekiewicz M., Załęski A., Albrecht P. (2016). Values from Three-dimensional High-resolution Anorectal Manometry Analysis of Children Without Lower Gastrointestinal Symptoms. Clin. Gastroenterol. Hepatol..

[8] Frye J., Rao S.S.C. (2024). Anorectal Manometry: When, How to Perform and Interpret, and Is It Useful?. Am. J. Gastroenterol..

[9] Scott S.M., Carrington E.V. (2020). The London Classification: Improving Characterization and Classification of Anorectal Function with Anorectal Manometry. Curr. Gastroenterol. Rep..

[10] Carrington E.V., Heinrich H., Knowles C.H., Fox M., Rao S., Altomare D.F., Bharucha A.E., Burgell R., Chey W.D., Chiarioni G. (2020). The international anorectal physiology working group (IAPWG) recommendations: Standardized testing protocol and the London classification for disorders of anorectal function. Neurogastroenterol. Motil..

[11] Banasiuk M., Dobrowolska M., Skowronska B., Konys J., Chorazyk A., Szudejko E., Banaszkiewicz A. (2021). Three-dimensional high-resolution anorectal manometry: Cut-off values for diagnosis of dyssynergic defecation in children. Eur. Rev. Med. Pharmacol. Sci..

[12] Sharma A., Misra A., Ghoshal U.C. (2014). Fecal Evacuation Disorder Among Patients with Solitary Rectal Ulcer Syndrome: A Case-control Study. J. Neurogastroenterol. Motil..

[13] Behera M., Dixit V., Shukla S., Ghosh J., Abhilash V., Asati P., Jain A. (2015). Solitary rectal ulcer syndrome: Clinical, endoscopic, histological and anorectal manometry findings in north Indian patients. Trop. Gastroenterol..

[14] Abbas T. (2011). Solitary Rectal Ulcer Syndrome, a Pediatric Problem: Case Report. Oman Med. J..

[15] Zhu Q.C. (2014). Solitary rectal ulcer syndrome: Clinical features, pathophysiology, diagnosis and treatment strategies. World J. Gastroenterol..

[16] Forootan M., Shekarchizadeh M., Farmanara H., Shekarchizadeh Esfahani A.R., Shekarchizadeh Esfahani M. (2018). Biofeedback efficacy to improve clinical symptoms and endoscopic signs of solitary rectal ulcer syndrome. Eur. J. Transl. Myol..

[17] Morio O., Meurette G., Desfourneaux V., D’Halluin P.N., Bretagne J.F., Siproudhis L. (2005). Anorectal Physiology in Solitary Ulcer Syndrome: A Case-Matched Series. Dis. Colon Rectum.

[18] Keighley M.R.B., Shouler P. (1984). Clinical and manometric features of the solitary rectal ulcer syndrome. Dis. Colon Rectum.

[19] Thirumal P., Sumathi B., Nirmala D. (2020). A Clinical Entity Often Missed—Solitary Rectal Ulcer Syndrome in Children. Front. Pediatr..

[20] Varol F.İ., Güngör Ş., Selimoğlu M.A., Şamdancı E. (2024). Rare Cause of Hematochezia in Children: Solitary Rectal Ulcer, Single Center Experience. Korean J. Gastroenterol..

[21] Perito E.R., Mileti E., Dalal D.H., Cho S., Ferrell L.D., McCracken M., Heyman M.B. (2012). Solitary Rectal Ulcer Syndrome in Children and Adolescents. J. Pediatr. Gastroenterol. Nutr..

[22] Hammad A., Aziz M.T., Khosa G.K., Sheikh M.A. (2022). Solitary rectal ulcer syndrome: Role of hydrocortisone enema in pediatric patients. Prof. Med. J..

[23] Dehghani S.M., Haghighat M., Imanieh M.H., Geramizadeh B. (2008). Solitary rectal ulcer syndrome in children: A prospective study of cases from southern Iran. Eur. J. Gastroenterol. Hepatol..

[24] Dehghani S.M. (2012). Solitary rectal ulcer syndrome in children: A literature review. World J. Gastroenterol..

[25] Blackburn C., McDermott M., Bourke B. (2012). Clinical Presentation of and Outcome for Solitary Rectal Ulcer Syndrome in Children. J. Pediatr. Gastroenterol. Nutr..

[26] Arı D., Öztürk Ö., Özin Y., Tenlik I., Bacaksız F., Gökbulut V., Kaplan M., Kalkan İ.H., Akdoğan M. (2022). The Efficacy of Biofeedback Therapy in Patients with Solitary Rectal Ulcer and Dyssynergic Defecation. Dig. Dis..

